# Intraoperative Computed Tomography, Ultrasound, and Augmented Reality in Mesial Temporal Lobe Epilepsy Surgery—A Retrospective Cohort Study

**DOI:** 10.3390/s25175301

**Published:** 2025-08-26

**Authors:** Franziska Neumann, Alexander Grote, Marko Gjorgjevski, Barbara Carl, Susanne Knake, Katja Menzler, Christopher Nimsky, Miriam H. A. Bopp

**Affiliations:** 1 Department of Neurosurgery, University Hospital Marburg, Philipps University Marburg, Baldingerstrasse, 35043 Marburg, Germany; franziska.neumann@uk-gm.de (F.N.); marko.gjorgjevski@uk-gm.de (M.G.); barbara.carl@helios-gesundheit.de (B.C.); nimsky@med.uni-marburg.de (C.N.); 2 Department of Neurosurgery, Helios Dr. Horst Schmidt Kliniken, Ludwig-Erhard-Straße 100, 65199 Wiesbaden, Germany; 3 Epilepsy Center Hesse, Department for Neurology, University Hospital Marburg, Philipps University Marburg, Baldingerstrasse, 35043 Marburg, Germany; knake@med.uni-marburg.de (S.K.); katja.menzler@med.uni-marburg.de (K.M.); 4 Center for Mind, Brain and Behavior (CMBB), 35043 Marburg, Germany; 5 LOEWE-Research-Cluster for Advanced Medical Physics in Imaging and Therapy (ADMIT), TH Mittelhessen University of Applied Sciences, 35390 Giessen, Germany

**Keywords:** epilepsy surgery, navigation, augmented reality

## Abstract

Mesial temporal lobe epilepsy (mTLE) surgery, particularly selective amygdalohippocampectomy (sAHE), is a recognized treatment for pharmacoresistant temporal lobe epilepsy (TLE). Accurate intraoperative orientation is crucial for complete resection while maintaining functional integrity. This study evaluated the usability and effectiveness of multimodal neuronavigation and microscope-based augmented reality (AR) with intraoperative computed tomography (iCT) and navigated intraoperative ultrasound (iUS) in 28 patients undergoing resective surgery. Automatic iCT-based registration provided high initial navigation accuracy. Navigated iUS was utilized to verify navigational accuracy and assess the extent of resection during the procedure. AR support was successfully implemented in all cases, enhancing surgical orientation, surgeon comfort, and patient safety, while also aiding training and education. At one-year follow-up, 60.7% of patients achieved complete seizure freedom (ILAE Class 1), rising to 67.9% at the latest follow-up (median 4.6 years). Surgical complications were present in three cases (10.7%), but none resulted in permanent deficits. The integration of microscope-based AR with iCT and navigated iUS provides a precise and safe approach to resection in TLE surgery, additionally serving as valuable tool for neurosurgical training and education.

## 1. Introduction

Augmented reality (AR) is currently a widely used technique in neurosurgery, allowing surgeons to overlay virtually acquired preoperative information onto their view of the patient [1]. This enhances and integrates standard image-guidance into the surgical field. The first introduction of virtual image overlays into the operating microscope’s optical view, as proposed by Kelly et al. [2] and Roberts et al. [3], laid a solid foundation for developing AR hardware in neurosurgical settings. Although initial AR hardware was limited, the emergence of head-up display (HUD) operating microscopes in the 1990s made AR support accessible to a broader neurosurgical audience [4,5]. Beyond standard image-guidance, which remains the gold standard, microscope-based AR is now frequently used in brain tumor surgeries. It allows for real-time visualization of tumors and vital structural and functional risk areas [6,7,8,9,10,11], thereby improving surgical orientation. Additionally, its availability has already been extended to other neurosurgical application areas such as skull base surgery [12] or vascular surgery [13].

Approximately 65 million individuals globally live with epilepsy, of which about one-third are classified as pharmacoresistant [14,15,16,17]. Patients with pharmacoresistant epilepsy, particularly, most frequently exhibit temporal lobe epilepsy (TLE) [18]. Today, for carefully selected patients within this group, resective surgery is a well-established treatment option that yields positive outcomes [18,19].

In the majority of TLE cases, seizures originate in the mesio-basal temporal structures, including the hippocampus, amygdala, and parahippocampal gyrus [20]. While the traditional approach has involved resecting the anterior temporal lobe (ATL) due to the critical function of the temporomesial structures in TLE, a more tailored method known as selective amygdalohippocampectomy (sAHE)—which preserves the temporal neocortex—has been introduced [20], both before the advent of navigation technology. Various surgical approaches have been described, including the transcortical, transsylvian, and subtemporal techniques, each offering distinct advantages [20,21,22,23,24]. However, as is generally the case in neurosurgery, the primary objective of resective surgery is to excise the targeted structure—in this case, the epileptogenic focus—without inducing new neurological deficits. Hence, comprehending the topography of the temporomesial target structures is crucial for the safe and effective selective resection of the amygdala, hippocampus, and parahippocampal gyrus [20,21].

Intraoperative image-guidance has demonstrated its advantages in epilepsy surgery by correlating imaging data, information obtained from multimodal diagnostics, and the patient’s anatomical specifics, which benefits safe and successful individualized resection [25,26,27,28,29]. Expanding beyond conventional navigation, which is typically performed using separate navigation displays close to the surgical field along with dedicated navigation instruments (e.g., pointer), microscope-based augmented reality (AR) offers immediate mapping of image data and patient anatomy. This technology virtualizes the physical instruments’ tip according to the microscope’s focal point, integrating all information into the surgical view. By eliminating the need to switch instruments or change viewing angles during surgery, microscope-based AR enhances the surgeon’s mental visualization of the imaging data, simplifying surgical orientation and the mapping of both imaging and anatomical data. This reduces attention shifts, thus improving surgeon comfort [1,30,31].

Tailored resection for mTLE such as sAHE with its different tissue saving approaches [21,32,33,34,35] in contrast to ATL is vital for pinpointing target structures and preventing an excessive approach in selected cases. In this case, in particular, accurate image-guidance combined with microscope-based AR might be highly advantageous as AR allows for direct operation on the visualized target structure, thereby minimizing the size of the access path. Furthermore, it might significantly reduce the risk of unintended mesial deviations, especially with transsylvian and transcortical accesses. The use of microscope-based AR support in temporal lobe epilepsy surgery has rarely been studied thus far. A recent report [36] investigated AR support in a pediatric epilepsy patient cohort with various entities and surgical strategies. However, despite showing the supportive effect of AR assistance, it did not elaborate on AR support in mTLE surgery, further tailoring resection and access paths, in clinical practice and education.

Consequently, this study seeks to report and evaluate the clinical experience, usability, practicality, and potential of microscope-based AR support in mTLE surgery, also for training and educational purposes. To date, reports focusing specifically on the use of neuronavigation and AR in relation to navigational accuracy essential for surgery and education in TLE surgery have been scarce.

## 2. Materials and Methods

### 2.1. Study Cohort

In this study, data from 28 patients with pharmacoresistant temporal lobe epilepsy who underwent resective surgery in the temporomesial lobe between September 2016 and December 2024 were analyzed. All patients underwent presurgical assessments following a standard protocol that included clinical, imaging, neuropsychological, and surface electroencephalography (EEG) data, as well as, in part, invasive EEG. This was followed by a surgical recommendation for selective temporomesial lobe resection by the interdisciplinary epilepsy surgery board (epileptology, neurosurgery, neuropsychology, neuroradiology).

Ethics approval for collecting routine clinical and technical data during the neurosurgical treatment of patients was obtained in accordance with the Declaration of Helsinki by the local ethics committee at the University of Marburg (No. 99/18); the analysis of the collected data was additionally permitted by the local ethics committee (24-214 RS). Written informed consent was provided by all included patients.

### 2.2. Preoperative Imaging and Planning

After initial diagnostic imaging and surgical recommendation, all patients underwent preoperative magnetic resonance imaging (MRI) using a 3T MRI system (Tim Trio, Siemens, Erlangen, Germany) equipped with a 12-channel head matrix Rx-coil. Data acquisition included a 3D T1-weighted, 3D T2-weighted, 3D Fluid Attenuated Inversion Recovery (FLAIR), time-of-flight (ToF) angiography data set, as well as a diffusion-weighted (DWI) single-shot echo-planar imaging (EPI) data set for fiber tractography with 30 non-collinear diffusion encoding gradients (high b-value 1000 s/mm^2^). If relevant, functional MRI (fMRI) data utilizing language tasks to localize Broca’s and Wernicke’s area were assessed to determine hemispheric lateralization.

Following rigid image co-registration of all data sets using the image fusion element (Brainlab, Munich, Germany), automatic segmentation of the amygdala, hippocampus, and brainstem, as well as the cerebrum, was performed using the anatomical mapping element (Brainlab, Munich, Germany) and manually refined if required. In addition, the lateral ventricle and vascular structures (middle cerebral artery) were segmented manually within the smart brush element (Brainlab, Munich, Germany).

Fiber tractography of the optic radiation and corticospinal tract was performed using the fiber tracking element (Brainlab, Munich, Germany) by that time based on the standard diffusion tensor imaging deterministic approach. If available, fMRI data sets were analyzed using SPM8/SPM12 in accordance with a standard processing protocol (without normalization). Resulting activation clusters (family-wise error corrected) were incorporated into the multimodal preoperative plan.

### 2.3. Operating Room Setup

All patients underwent microscope-based AR-supported resection of temporomesial structures (amygdala, hippocampus) using navigation and microscope technologies in cases of unilateral TLE. The operating room is equipped with an optical neuronavigation system (Curve Navigation, Brainlab, Munich, Germany), a mobile 32-slice intraoperative CT (iCT) system (AIRO^®^, Brainlab, Munich, Germany) for registration and control scans, operating microscopes (Pentero 900 or Kinevo 900, Zeiss, Oberkochen, Germany), and ultrasound systems (FlexFocus 800/BK5000, BK Medical, Herlev, Denmark), both fully integrated into the navigation system with a convex craniotomy transducer, respectively.

### 2.4. Intraoperative Workflow

**Figure 1:**
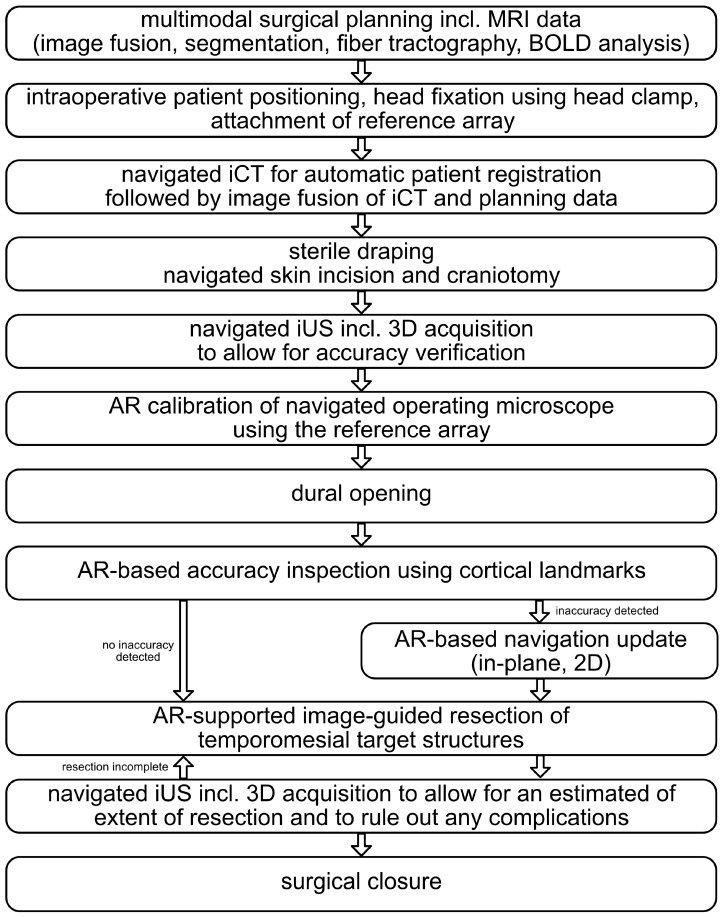
Overall workflow of the presented approach to MTLE surgery investigating intraoperative computed tomography (iCT), navigated intraoperative ultrasound (iUS), and augmented reality (AR) support.

An overview of the overall workflow is provided in Figure 1, and a detailed description of all procedural steps can be found in the following.

Under general anesthesia, the patient’s head was fixated in a radiolucent carbon head clamp (DORO, Black Forest Medical Group, Freiburg, Germany) using three metallic pins with those pins placed outside the area of interest to prevent artifacts in a potential full-dose control scan at the end of resection. To allow for navigational support, a radiolucent patient reference geometry was mounted on the left side of the head clamp. To assess registration accuracy, three adhesive skin markers were attached to the patient’s head within the scan area.

A sequential low-dose iCT scan with limited scan length was performed for automatic intraoperative patient registration (7.1 mA, 120 kV, 1.92 s exposure time, 1 mm reconstructed slice thickness, 512 × 512 matrix size, 33.3 cm^2^ field of view, 6.2 cm scan length, resulting in a dose-length product of 17.8 mGy*cm). The target registration error (TRE) was estimated using the attached skin markers as offset between the physical pointer’s tooltip (Cranial Pointer, Brainlab, Munich, Germany) that was placed in the divot of the three skin markers and the tooltip in the virtual representation of the skin markers within the generated iCT data set. After assuring high patient registration using the attached skin markers, the preoperative data including the surgical plan was rigidly co-registered with the low-dose registration iCT scan, allowing instant navigation support.

Following a standardized skin incision, a navigation-supported tailored craniotomy was performed to assess the temporomesial target structures. Before durotomy, navigated ultrasound (iUS) imaging was performed using a trackable craniotomy transducer (8862/N13C5, BK Medical, Herlev, Denmark) with standard settings (imaging depth 63 mm/65 mm, B-mode) to (1) assess navigation accuracy by overlaying segmented outlines based on the preoperative MRI data onto the live-ultrasound view and (2) to identify the temporomesial target structures and landmarks within the ultrasound data for additional guidance and later intraoperative resection control. Therefore, in addition to the navigated live-ultrasound usage, a 3D iUS data set was acquired by uniformly sweeping the transducer across the accessible dural layer within the craniotomy. Further, a vascular 3D iUS data set (C-Mode) was acquired analogously to allow for visualization of relevant vascular structures.

Besides navigation support in terms of pointer-based navigation with navigation displays close by the surgical field with fused multimodal image data enriched with the outlined objects and structures, microscope-based navigation using augmented reality was enabled using the microscope navigation element (Brainlab, Munich, Germany). To enable this, the head-up displays (HUDs) of the operating microscopes Pentero 900/Kinevo 900 (Zeiss, Oberkochen, Germany), fully integrated into the navigational setup, were utilized. The microscope was tracked (Optical Tracking System, Brainlab, Munich, Germany) in the navigational space using an attached 4-sphere reference array to allow for AR support throughout the surgery. Before usage, the AR visualization was calibrated to reduce minor spatial initial misalignments using the reference array. Based on that, all outlined objects such as the hippocampus, the amygdala, the lateral ventricle, the brainstem, the vascular tree, the optic radiation, and the corticospinal tract could be visualized utilizing the HUD of the operating microscope within surgical view enabling microscope-based AR support throughout the whole microsurgical part of the procedure. The objects can be visualized using the HUD either in 2D (solid object outlines representing the diameter in the recent focal plan with dotted outlines displaying the maximal object extend beyond) or 3D fashion. However, to adapt to the surgeon’s needs and the surgical phase, all included object outlines can separately be switched on and off at any time to provide the best support.

After the dural opening, the navigation accuracy was reassessed using the outlined cortical profile. If there were any slight in-plane deviations (2D, no depth inaccuracies), the augmented overlay was adjusted by translating and rotating it to better fit the patient’s anatomy. Accurate neuronavigation and microscope-based AR allow for direct operation on the target structure. Once the middle temporal gyrus was located, the cortex was incised minimally based on image-guidance, and dissection was performed towards the temporal horn, which serves as a major anatomical landmark, with image-guidance reducing the risk of unintended mesial deviations. Upon entering the temporal horn, self-retaining brain spatulas were inserted to provide an optimal view. In the initial step, the parahippocampal gyrus was examined and dissected in a temporo-basal direction, ensuring that the medial arachnoidal border was preserved. After the subpial resection of the uncus, the resection was extended medially and posteriorly to remove the hippocampus, preferably en bloc or as anterior and posterior samples. Subsequently, the amygdala was resected, despite the absence of clear anatomical borders.

Following resection of the temporomesial target structures, navigated intraoperative ultrasound (iUS) was re-evaluated to (1) confirm and assess the extent of the resection while in surgery and (2) rule out any complications along the resection cavity. To achieve this, a 3D iUS data set was obtained by consistently sweeping the transducer over the saline-filled resection cavity.

### 2.5. Epileptogenic Outcome

Epileptogenic outcome was postoperatively assessed according to the International League Against Epilepsy (ILAE) outcome classification [37] at on-site follow-up examinations one year after surgery and at the most recent follow-up. Complete seizure-freedom (ILAE 1) was considered an excellent epileptogenic outcome, while at latest follow-up, ILAE classes 1, 2, and 3 were considered a favorable outcome [38].

Assessment of functions of the dominant and non-dominant mesial temporal lobe with the delayed recognition trial of the verbal learning and memory test (VLMT [39]) assessing verbal declarative memory functions (encoding/decoding) as well as the delayed recall of the Rey–Osterrieth Complex Figure Test (ROCFT [40]) for non-verbal memory function were performed preoperatively and during follow-up.

## 3. Results

### 3.1. Clinical and Demographic Information

In this study, data from 28 patients (mean age: 39.75 ± 15.49 years, male/female: 14/14) who underwent surgery for mTLE were analyzed. Twelve patients underwent selective amygdalohippocampectomy on the right side, and fourteen on the left side; in two additional cases, only resection of the amygdala was performed. Neuropathological examination of the resected tissue revealed hippocampal sclerosis (*n* = 22), heterotopia (*n* = 2), gliosis (*n* = 2), dysembryoplastic neuroepithelial tumor (*n* = 1), and glioma (*n* = 1).

### 3.2. Epileptogenic Outcome

**Figure 2:**
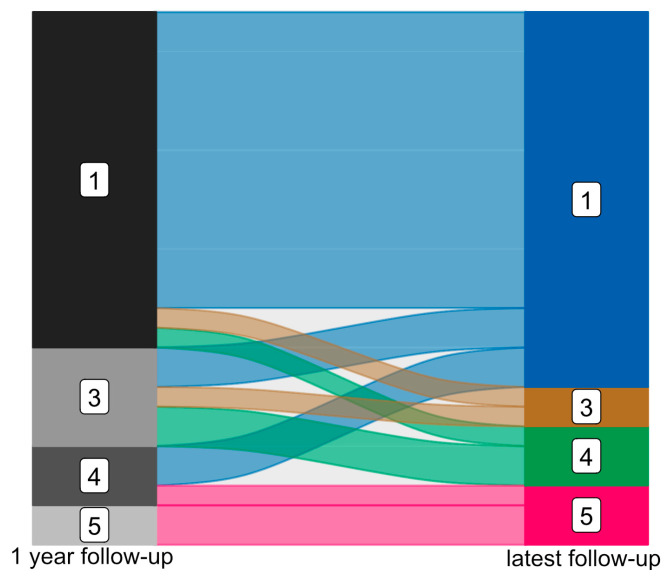
Epileptogenic outcome after mTLE surgery after 1 year (**left**) and at latest available follow-up (**right**) classified according to ILAE (ILAE class 1 to ILAE class 5).

At the one-year follow-up, 17 patients (60.71%) were categorized as ILAE class 1, with no seizures reported after resective surgery. Five patients were assigned to ILAE class 3 (17.86%), three to ILAE class 4 (10.71%), and two to ILAE class 5 (7.14%). One patient (3.57%) did not undergo any postoperative follow-up examination. In a long-term follow-up with varying intervals, 19 patients (67.86%) remained seizure-free at their most recent follow-up (median: 4.62 years, interquartile range: 2.93), classified as ILAE class 1. Two patients were classified as ILAE class 3 (one had deteriorated from ILAE class 1), while three were categorized as ILAE class 4 (two had deteriorated from class 3 and one from class 1), and three patients were termed ILAE class 5 (one had deteriorated from class 4, the others remained in class 5). At the latest follow-up, 75.00% of patients exhibited favorable outcomes, with 67.86% completely seizure-free. A slight reduction in the initial group classified in ILAE class 1 was noted (two patients), while four patients remained seizure-free during long-term follow-up despite having auras or seizures at the one-year mark; see Figure 2.

Neuropsychological and neurocognitive assessments were available pre- and postoperatively were available in 21 cases (seven patients did not participate in preoperative or follow-up assessments; one patient was excluded due to age (<5 years)). For patients who underwent MTLE surgery on the dominant side (*n* = 11) results of the delayed recognition trial of the VLMT revealed means of 9.9 ± 4.0 vs. 8.0 ± 4.8 for preoperative and follow-up assessments. In case of patients with MTLE surgery on the non-dominant side (*n* = 10), results of the delayed recall of the ROCFT revealed means of 13.9 ± 5.2 vs. 17.5 ± 6.4 for preoperative and follow-up assessments.

### 3.3. Complications

Among the twenty-eight patients who underwent resection, four cases of surgical complications were observed. Two patients developed a subgaleal cerebrospinal fluid (CSF) fistula, which was managed with lumbar CSF drainage for five days. After the drainage was removed, no further intervention was required. Another patient presented with an abscess that necessitated surgical intervention. In the final case, the patient experienced a remote cerebellar hemorrhage, which was addressed with suboccipital decompression and external ventricular drainage. Following these procedures, no additional surgeries were necessary.

Five patients who had surgery in the dominant temporal lobe developed transient postoperative aphasia, which resolved completely by the time of discharge. One patient experienced postoperative oculomotor paralysis, which fully improved within four weeks after surgery.

Postoperative visual field deficits (VFDs) were observed in seven cases; all seven cases were confirmed to have quadrantanopia through computerized visual field perimetry. Nine patients did not exhibit any VFD, while in the remaining eleven patients, the examination could not be successfully performed due to compliance issues.

### 3.4. Navigation and Augmented Reality Support

Navigation and microscope-based AR support, enabled by automatic iCT-based registration and intraoperative ultrasound, was facilitated in all surgeries with a mean initial TRE of 0.74 ± 0.28 mm. In all cases, visualization of target structures and risk structures related to the surgical trajectory or in proximity of the surgical target was provided in the surgical plan and enhanced the microscopic view throughout the surgery. Provided visualizations included outlines of the brainstem (risk structure), amygdala and hippocampus (target structures), lateral ventricle (landmark), carotid arteria and media (risk structures/landmark), cortical vessels and cerebrum (verification/navigation update) and fiber tractography of the optic radiation (risk structure). To provide efficient AR support, tailored to the surgeon’s preferences and needs, all structures could be separately switched on and off. In this way, microscope-based AR support allowed for improved intraoperative orientation for the surgeon but also the assisting surgeon (residents) and the OR staff using the microscopic view displayed on the navigation screens close by, and therefore contributed to patient safety while in parallel increasing surgeon’s comfort.

### 3.5. Workflow Illustrations

**Figure 3:**
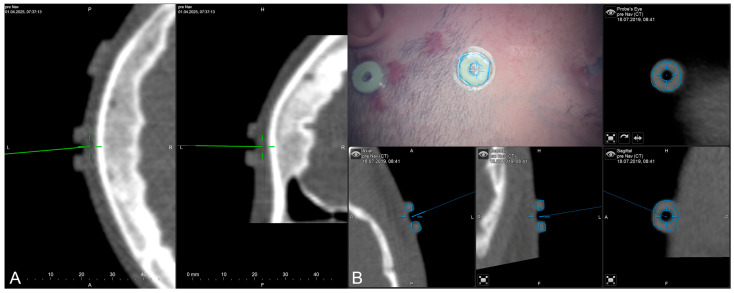
Accuracy checks using artificial landmarks either assessed with a pointer (**A**) or the virtualized pointer using the operative microscope (**B**) with segmented outlines of the artificial marker visualized in the operating field using microscope-based AR.

The applicability of navigation and microscope-based AR support during the surgical procedure and educational purposes tremendously depends on the navigational accuracy. The presented setup allowed for different methods to gain, keep, and restore navigation accuracy throughout the procedure. In the recent setup, automatic intraoperative CT-based registration is used for mapping of image and patient data showing a high registration accuracy with very low TREs estimated using artificial landmarks. Navigation accuracy can be verified and quantified in different ways throughout the procedure, e.g., using artificial landmarks not used for the registration procedure assessed with a pointer (see Figure 3A) or using microscope-based AR with segmented outlines of the landmarks (see Figure 3B).

**Figure 4:**
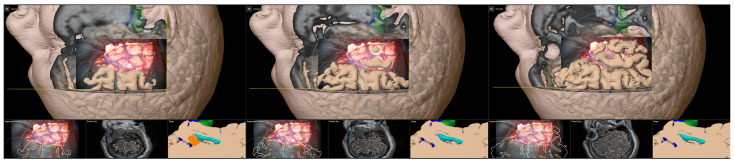
Accuracy checks using a 3D reconstruction of the cortical profile with the microscope’s focal plane being stepwise moved along the viewing trajectory.

Furthermore, during the surgical procedure, AR can also be utilized for accuracy checks and navigation updates to reassure high navigational accuracy throughout the intervention using anatomical landmarks, such as cortical vascular structures eligible for in-plane (2D, no depth inaccuracies) corrections of spatial inaccuracies (for further details see [41]) as well as 3D reconstruction of the cortical profile (see Figure 4).

**Figure 5:**
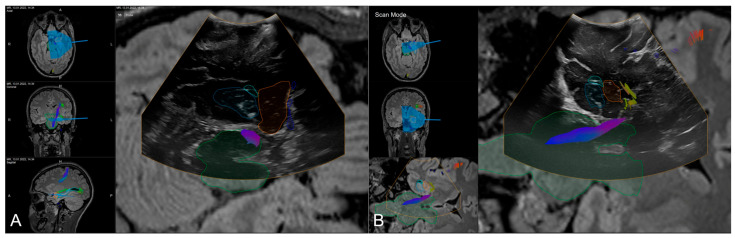
Navigated intraoperative ultrasound allowing for accuracy checks with outlined MRI-based objects (orange: amygdala, blue: hippocampus, green: brainstem, light blue: ventricle) and fiber tractography of the corticospinal tracts and optic radiation in axial (**A**) and coronar (**B**) view.

**Figure 6:**
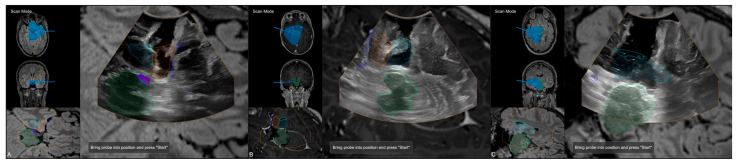
Navigated intraoperative ultrasound after resection of the amygdala (orange) and hippocampus (blue) visualizing the extent of resection and surgical trajectory in axial views (**A**,**B**) and oblique view (**C**).

In addition, as described in the workflow, navigated intraoperative ultrasound can be investigated to verify navigation accuracy (see Figure 5) before resection using the live view or acquiring a 3D iUS data set, as well as to estimate intraoperative extent of resection and exclusion of intraoperative complications (see Figure 6).

**Figure 7:**
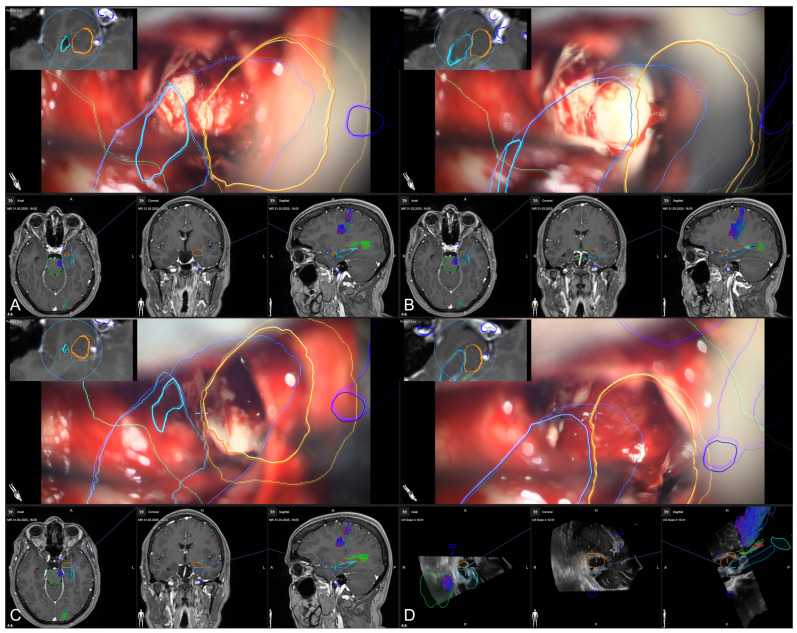
Microscope-based augmented reality and image-guidance in the course of surgery while assessing the temporal horn/hippocampus (**A**), the hippocampus (**B**), parts of the amygdala (**C**), and finally, showing the arachnoid level after resection of the amygdala (**D**) with preoperative MRI data (**A**–**C**) as well as intraoperative US data after resection (**D**) in the navigation panel below the microscopic view in axial, coronal, and sagittal orientation, as well as a probe’s eye view (left upper corner) of preoperative MRI data according the recent microscope’s focal plane, allowing for immediate matching of imaging data and patient’s anatomy (blue: hippocampus, orange: amygdala, green: brainstem, light blue: temporal horn, dark blue: vessels).

In the course of resection, image-guidance is provided on a screen close-by with the microscope’s focal point used as virtual pointer. In parallel, the outlines of all segmented structures (e.g., hippocampus, temporal horn, amygdala, brainstem) are visualized within the microscopic view, allowing for an immediate transfer of imaging data into the surgical situs (see Figure 7).

## 4. Discussion

This study aimed to showcase both the clinical benefits and the educational potential of neuronavigation, particularly microscope-based augmented reality (AR) support, in surgery for mTLE. In all cases, microscope-based AR assistance was employed, enhancing intraoperative surgical orientation, patient safety, and surgeon comfort. Consequently, the findings of this study bolster the hypothesis regarding the educational benefits and clinical advantages of utilizing microscope-based AR support in treating patients with temporal lobe epilepsy.

Resective surgery is a well-established treatment option for carefully selected patients with pharmacoresistant epilepsy, primarily those with TLE [18,19]. Surgical treatment, particularly for mTLE, is regarded as safe and standardized, offering advantages over conservative approaches [18]. A crucial factor for successful surgical intervention is the precise identification of the epileptogenic zone [38]. In most cases of TLE, seizure origins are located in the mesio-basal temporal structures, such as the hippocampus, amygdala, and parahippocampal gyrus, which are typically resected using a selective approach (sAHE) that spares the temporal neocortex [20]. While various techniques exist, including transcortical, transsylvian, and subtemporal approaches, a deep understanding of the anatomy of the temporomesial target structures, surrounding risk areas, and intraoperative landmarks is essential for the safe and successful selective resection of the amygdala, hippocampus, and parahippocampal gyrus [20,21,22,23,24].

While having extensive knowledge of the topography and significant landmarks can guide surgical procedures without the need for neuronavigation, the technology remains a valuable addition. It aids in pinpointing the cortical incision’s location, directing dissection toward the temporal horn, and identifying key target structures for intraoperative orientation. This helps to avoid unintended trajectories that are too mesial [20,35]. Additionally, neuronavigation support allows surgeons to accurately assess the extent of resection during the operation [35], as demonstrated in this study, which utilized both standard navigation and real-time imaging techniques like navigated ultrasound.

Nevertheless, comprehensive surgical planning is essential, particularly in understanding the spatial relationships between target structures and nearby risk areas along the surgical route. This understanding not only enhances preoperative and intraoperative decision-making but also assists in surgical orientation and contributes to more radical resections while improving patient safety [42,43,44,45,46,47]. Consequently, neuronavigation, along with microscope-based AR support, has been integrated into functional and epilepsy surgeries for treatment planning, invasive diagnostics, and the resection of epileptogenic zones [25,26,27,29,48,49,50].

Standard navigation typically relies on dedicated instruments and separate displays situated near the surgical field. This arrangement necessitates frequent switching of instruments and changing viewing angles between the patient and display during surgery [31], often requiring the surgeon to mentally synchronize the navigation data with their understanding of the surgical site, which heavily relies on their experience. Microscope-based AR enhances mental visualization by integrating navigational tooltips into the microscope’s focal point, combining all navigational information into the surgical view. This approach improves orientation, reduces the need for attention shifts, and increases the comfort of the surgeon [1,30,31]. It also offers opportunities to enhance mapping image data and understand the patient’s anatomy, facilitating the identification of key landmarks for intraoperative awareness and spatial relationships between targets and risk structures relevant to specific surgical approaches. Early versions of microscope-based AR displayed manually outlined objects using dashed and solid lines in the current focal plane through the microscope’s HUD [10,51,52]. Although 2D representations may limit depth perception [53], the latest state-of-the-art implementations enable better 3D perception of outlined structures overlaid onto the surgical view. Enhanced HUD resolution, the incorporation of multiple colors for object differentiation, and smooth real-time visualization—made possible by significant advances in computing power—support a more intuitive use of microscope-based AR. The complexity of the visualization and the variety of visualizable objects can be individually toggled to prevent clutter in the surgical view, adapting to each surgeon’s needs and the current surgical phase. Thus, microscope-based AR is viewed as an addition, complementing standard navigation, as it can provide contextual information beyond what is currently visualized [1,31].

While augmented reality based on navigation and microscopy offers a distinct advantage for intraoperative surgical orientation and educational purposes—by aligning in-situ surgical landmarks with enhanced imaging data—its effectiveness relies heavily on accurately matching patient and imaging spaces; misalignment can lead to misleading and potentially dangerous situations if one solely depends on this technology. Consequently, achieving high navigational and overall accuracy is essential. Overall accuracy is a multifaceted term, influenced by four key domains: imaging accuracy, technical accuracy, registration accuracy, and the impact of intraoperative events, all contributing to application accuracy [43,54].

Imaging accuracy pertains to the specific imaging modality, like MRI, which has inherent benefits, such as tissue contrast, and drawbacks, including non-linear geometric distortions that complicate image fusion with non-distorted imaging data like CT. This is particularly significant during stereotactic procedures, such as electrode placement or biopsies [46,55,56].

Technical accuracy, on the other hand, involves the intrinsic precision of the navigation system, including tracking technology and the geometric integrity of the various instruments used (e.g., microscope, ultrasound probe, pointer) [57], which has demonstrated less than 3 mm accuracy in frameless setups [54].

Registration accuracy is now recognized as a crucial factor affecting application accuracy, with various methods available [58,59,60]. In frameless setups, patient registration is commonly conducted using either landmark-based or surface-based approaches. The landmark-based method utilizes anatomical landmarks, but it often employs artificial markers placed across the patient’s head to facilitate 3D paired point rigid registration between imaging and real-world markers [54]. However, this method has demonstrated varying target registration errors (TREs) ranging from 1.8 mm to 5.0 mm [58,61], influenced by factors such as the number of markers, their positioning, spatial arrangement, skin shifts during imaging (e.g., caused by padding or protective headphones), and intraoperative conditions (e.g., skin displacement due to three-pin fixation or the pointer during registration), as well as the user [54,62,63,64,65]. Similarly, surface-based methods integrate anatomical landmarks with laser surface matching to allow for a virtual sampling of the patient’s head for registration. Despite not being dependent on specific preoperative imaging with adhesive skin markers, its application is limited due to image quality and intraoperative patient positioning, with reports indicating even lower registration accuracy—mean TREs of 5.3 mm—depending on the imaging modality and quality [59]. The advent of intraoperative CT and MRI imaging has enabled automatic and user-independent registration achieving TREs of around or less than 1 mm [41,48,60,62], as also seen in this study with a mean TRE of 0.74 ± 0.28 mm. This registration procedure, as a key component, significantly reduces errors introduced at this stage of surgery prior to skin incision, thereby enhancing overall clinical accuracy when compared to other widely used methods. It provides an ideal foundation for surgical orientation and educational purposes while aligning imaging and augmented reality (AR) data with the patient’s anatomy.

Surgical navigation accuracy is known to continuously diminish during procedures due to factors such as the application of surgical drapes, skin incisions, trepanation, craniotomy, and the length of surgery [42,58,66]. This decline primarily results from changes in the spatial relationship between the patient’s head and the reference array. Although brain shift may be minimal before the dural opening, the non-linear deformations of the brain can occur in unpredictable ways due to factors like the use of brain retractors, loss of cerebrospinal fluid (CSF), gravitational effects, and ongoing tissue resection, all contributing to unavoidable errors. Consequently, intraoperative accuracy post-registration is compromised, hindering the direct and intuitive connection of virtual data to real-world surgical orientation and education, which in severe cases can render navigation ineffective. Thus, it is crucial to (1) recognize potential intrinsic and extrinsic inaccuracies that may arise during the procedure and (2) be equipped to identify and potentially correct these inaccuracies.

Multiple strategies have been established to tackle this challenge, including intraoperative imaging methods like MRI [44,45,67,68] or ultrasound [69,70,71]. These techniques can either update planning and navigation data with intraoperative data or convert complex preoperative data to reflect the recent intraoperative situation in a non-linear manner. The use of iMRI and repetitive iMRI is restricted due to limited availability, structural demands, prolonged time requirements, and high costs. As a result, these methods are more often employed for resection control in neurooncology or for navigation updates later in surgery when significant brain shift occurs. In contrast, intraoperative ultrasound can be utilized at any point during the surgical procedure and can be repeated without noticeable time delays. It is cost-effective, especially when seamlessly integrated into the neuronavigation system. While non-linear image fusion using iMRI data remains a long-term research focus and is clinically available [68], non-linear image registration based on intraoperative ultrasound is still in development. Recently, clinically established rigid image fusion of preoperative MRI and intraoperative ultrasound data has facilitated local navigation updates [72]. Notably, in addition to the intraoperative imaging techniques discussed earlier and exemplified in this study, microscope-based AR offers a convenient way to monitor navigation accuracy by examining uniquely identifiable landmarks and structures—like cortical vessels, the cortex profile, bony landmarks when relevant, and deep vascular structures [41]. It also compensates for in-plane registration inaccuracies, assuming high calibration precision of the microscope [73]. However, current AR adjustments are still confined to translational and rotational transformations within the microscope’s focal plane (2D adjustment). Even though accuracy checks can be performed at various points in the surgical site, AR adjustments still have limitations; as recently implemented, AR base adjustments can only compensate for in-plane, 2D misalignments, rather than 3D inaccuracies or depth misalignments and are therefore not yet fully capable of overcoming all kinds of misalignments. Nevertheless, microscope-based AR support enables accuracy monitoring and navigation updates in subcortical areas, provided uniquely identifiable landmarks such as vascular structures are present [13].

While microscope-based AR is commonly utilized in neurosurgery, the virtualization added to the microscopic view introduces a further element to the accuracy chain [73]. The accuracy of AR entails both the inherent precision of the AR system, which cannot be modified by the user, and the user-dependent calibration of AR within the surgical environment. This approach ensures high levels of AR and navigation accuracy at this stage.

Alongside its clinical benefits of microscope-based AR by offering assistance during surgery, AR has also emerged as a valuable tool for neurosurgical education and training [74]. In the educational setting, AR is most often used to enable simulations to create a virtual risk-free environment for neurosurgical trainees to develop and refine their surgical skills repetitively, and is seen as an important adjunct to training on real patients [75,76]. The first setups included training material for external ventricular drainages (EVD) and needle biopsies with printed skull phantoms, and AR demonstrated its ability to aid in EVD placement and needle biopsy insertion [77]. Furthermore, AR has been utilized in a training setup for locating, navigating, and rating malignant brain lesions, e.g., for craniotomy planning and surgical trajectory training [78] or delineation of tumor borders [79]. However, in vitro training setups are somewhat limited and unable to fully simulate a real patient’s anatomy. Newer investigations are incorporating haptic technology into simulators to replicate a real surgical experience and synthetic tissue simulations [80,81]. Despite these tremendous developments, simulations still cannot provide an entirely realistic experience for trainees, as it would be of utmost importance for surgeons practicing medicine on real patients [74]. Therefore, simulations must be designed in a way that creates a realistic visual and tactile experience to maximize the educational advantage.

In this way, training and education should be based on various adjuncts. Besides AR simulators for procedural training, AR can also be used, as shown in this study in terms of microscope-based AR with high navigational accuracy, to support education and training for residents in matching image data and real patient anatomy in a real surgical setting during surgery but also by using video recordings of navigation and in-parallel microscope data for offline training. This provides the potential to identify surgical landmarks and trajectories to support surgical orientation and mental representation of virtual image content connected to the patient’s anatomy, especially in cases of a limited number of patients with specific pathologies. Therefore, microscope-based AR represents a valuable adjunct to AR-based simulations, extending the surgical education toolbox.

The presented surgical workflow includes various technical instruments added to the standard clinical image-guided navigation approach, all supporting and contributing to its valuable use. One major prerequisite is the best possible mapping of imaging and patient data to ensure reliable and trustworthy image-guidance and AR support. As mentioned earlier, applying iCT for automatic patient registration allows for highly accurate, user-independent matching of imaging and patient data at the beginning of the procedure. Therefore, it serves as a highly accurate navigation initialization. To ensure high navigation accuracy before assessing the brain itself after dural operating, intraoperative imaging such as navigated iUS can be utilized to verify accuracy, estimate the amount of inaccuracy, or recently, also allowing for navigation updates by iUS-iMRI image fusion to overcome misalignments. With the latter, one might also overcome initial registration inaccuracies. Additionally, iUS can serve as a tool to determine the extent of resection intraoperatively and rule out surgical complications around the resection cavity. As shown here, microscope-based AR might also contribute to navigational accuracy by allowing for minor alignments based on AR and patient anatomy at various stages of surgery. In addition to accurate image-guidance supported by iCT and/or iUS, microscopes with AR enhance the surgical view with contextual information. This allows for immediate surgical orientation without the need to constantly mentally transfer 2D imaging data onto the surgical site. It provides further intuitive surgical guidance for tailored resection in close proximity to risk structures, given high navigational accuracy. However, achieving high navigation accuracy independently of image-guidance, with or without AR support, is a key prerequisite. This should be pursued using the technical tools available to make full use of the benefits of image-guidance with or without AR support.

The limitations of this study include its retrospective design and the moderate sample size, which results from the selection criteria that exclusively recruited TLE patients who underwent selective temporomesial resection. These criteria facilitate a more nuanced examination of microscope-based AR support within clinical and educational framework related to this particular procedure. However, while the sample size is reasonable for a technical study, it limits broader generalizability. Therefore, multicenter studies are required to confirm the results presented here. In addition, due to its retrospective nature, comparisons with standard techniques (e.g., image-guidance only) are lacking, necessitating future controlled trials to further elaborate on the additional value of AR assistance. Another limitation of the retrospective analysis is the lack of objective, quantifiable measures of the benefits in terms of surgical orientation and comfort, as well as a lack of prospectively collected trainee assessments to quantify the educational benefits further. This shall be overcome in future prospective analyses using trainee and surgeon assessments.

However, in light of the well-documented benefits of these techniques across the broader spectrum of neurosurgery, specifically in enhancing resection precision, increasing patient safety, and improving functional outcomes, as well as providing pedagogical guidance, there exists a critical need to engage in discussions regarding the implications of not adopting these methodologies.

## 5. Conclusions

Mesial temporal lobe epilepsy (MTLE) surgery, including selective amygdalohippocampectomy, is a well-established treatment for patients with drug-resistant TLE. However, precise intraoperative orientation is crucial for the safe and complete removal of epileptogenic structures while preserving functional integrity. Microscope-based AR support relying on high navigational accuracy enhances surgical orientation, even for experienced surgeons, and provides ergonomic comfort, ultimately increasing patient safety. Additionally, microscope-based AR serves as a valuable tool for neurosurgical training and education by creating a mental representation of the structural and functional anatomy of the patient and corresponding imaging data. This technology helps to identify key surgical and procedural landmarks. Despite the study’s retrospective design and moderate sample size, it highlights the importance of adopting such technologies to achieve surgical excellence and improve training in complex epilepsy procedures.

## Data Availability

The data in this study are available on request from the corresponding author. The data are not publicly available due to privacy restrictions.
